# Supplementary material for: Iterative improvement in the automatic modular design of robot swarms
Source: PeerJ Comput Sci. 2020 Dec 7;6:e322. doi: 10.7717/peerj-cs.322 (PMC7924708; doi:10.7717/peerj-cs.322)
Supplement: Supplemental Information 3 [file peerj-cs-06-322-s003.zip › argos3/doc/api/standalone/a00652_source.html]

ARGoS: plugins/simulator/visualizations/qt-opengl/qtopengl\_lua\_syntax\_highlighter.cpp Source File


- Main Page
- Related Pages
- Namespaces
- Classes
- Files

- File List
- File Members

# plugins/simulator/visualizations/qt-opengl/qtopengl\_lua\_syntax\_highlighter.cpp

Go to the documentation of this file.

```
00001 
00007 #include "qtopengl_lua_syntax_highlighter.h"
00008 
00009 namespace argos {
00010 
00011    /****************************************/
00012    /****************************************/
00013 
00014    static int NOT_MULTILINE_COMMENT = 0;
00015    static int MULTILINE_COMMENT = 1;
00016 
00017    /****************************************/
00018    /****************************************/
00019 
00020    CQTOpenGLLuaSyntaxHighlighter::CQTOpenGLLuaSyntaxHighlighter(QTextDocument* pc_text) :
00021       QSyntaxHighlighter(pc_text) {
00022       SHighlightingRule sRule;
00023       m_cKeywordFormat.setForeground(Qt::darkBlue);
00024       m_cKeywordFormat.setFontWeight(QFont::Bold);
00025       QStringList cKeywordPatterns;
00026       cKeywordPatterns << "\\band\\b"    << "\\bbreak\\b"  << "\\bdo\\b"   << "\\belse\\b"     << "\\belseif\\b"
00027                        << "\\bend\\b"    << "\\bfalse\\b"  << "\\bfor\\b"  << "\\bfunction\\b" << "\\bif\\b"
00028                        << "\\bin\\b"     << "\\blocal\\b"  << "\\bnil\\b"  << "\\bnot\\b"      << "\\bor\\b"
00029                        << "\\brepeat\\b" << "\\breturn\\b" << "\\bthen\\b" << "\\btrue\\b"     << "\\buntil\\b" << "\\bwhile\\b";
00030       foreach (const QString& cPattern, cKeywordPatterns) {
00031          sRule.Pattern = QRegExp(cPattern);
00032          sRule.Format = m_cKeywordFormat;
00033          m_vecHighlightingRules.append(sRule);
00034       }
00035 
00036       m_cQuotationFormat.setForeground(Qt::darkGreen);
00037       sRule.Pattern = QRegExp("\".*\"");
00038       sRule.Format = m_cQuotationFormat;
00039       m_vecHighlightingRules.append(sRule);
00040 
00041       m_cSingleLineCommentFormat.setForeground(Qt::darkGray);
00042       m_cSingleLineCommentFormat.setFontItalic(true);
00043       sRule.Pattern = QRegExp("--[^[\n]*");
00044       sRule.Format = m_cSingleLineCommentFormat;
00045       m_vecHighlightingRules.append(sRule);
00046 
00047       m_cMultiLineCommentFormat.setForeground(Qt::darkGray);
00048       m_cMultiLineCommentFormat.setFontItalic(true);
00049       m_cCommentStartExpression = QRegExp("--\\[\\[");
00050       m_cCommentEndExpression = QRegExp("\\]\\]");      
00051    }
00052 
00053    /****************************************/
00054    /****************************************/
00055 
00056    void CQTOpenGLLuaSyntaxHighlighter::highlightBlock(const QString& str_text) {
00057       /*
00058        * Apply normal rules
00059        */
00060       foreach (const SHighlightingRule& sRule, m_vecHighlightingRules) {
00061          QRegExp cExpression(sRule.Pattern);
00062          int i = cExpression.indexIn(str_text);
00063          while(i >= 0) {
00064             int nLength = cExpression.matchedLength();
00065             setFormat(i, nLength, sRule.Format);
00066             i = cExpression.indexIn(str_text, i + nLength);
00067          }
00068       }
00069       /*
00070        * Apply multi-line comment rules
00071        */
00072       setCurrentBlockState(NOT_MULTILINE_COMMENT);
00073       int nStartIndex = 0;
00074       if (previousBlockState() != MULTILINE_COMMENT) {
00075          nStartIndex = m_cCommentStartExpression.indexIn(str_text);
00076       }
00077       while(nStartIndex >= 0) {
00078          int nEndIndex = m_cCommentEndExpression.indexIn(str_text, nStartIndex);
00079          int nCommentLength;
00080          if (nEndIndex == -1) {
00081             setCurrentBlockState(MULTILINE_COMMENT);
00082             nCommentLength = str_text.length() - nStartIndex;
00083          } else {
00084             nCommentLength = nEndIndex - nStartIndex
00085                + m_cCommentEndExpression.matchedLength();
00086          }
00087          setFormat(nStartIndex, nCommentLength, m_cMultiLineCommentFormat);
00088          nStartIndex = m_cCommentStartExpression.indexIn(str_text, nStartIndex + nCommentLength);
00089       }
00090    }
00091 
00092    /****************************************/
00093    /****************************************/
00094 
00095 }
```

---

Generated on 10 Jul 2018 for ARGoS by 
 1.6.1
